# Association of hypertension in pregnancy with serum electrolyte disorders in late pregnancy among Cameroonian women

**DOI:** 10.1038/s41598-023-47623-6

**Published:** 2023-11-28

**Authors:** Atem Bethel Ajong, Martin Ndinakie Yakum, Loai Aljerf, Innocent Mbulli Ali, Fulbert Nkwele Mangala, Ukaogo Prince Onydinma, Blaise Mbuomboh Liwo, Cavin Epie Bekolo, Theodore Yangsi Tameh, Bruno Kenfack, Phelix Bruno Telefo

**Affiliations:** 1 Kekem District Hospital, Kekem, West Region Cameroon; 2 Department of Biochemistry, University of Dschang, Dschang, West Region Cameroon; 3 Department of Epidemiology and Biostatistics, School of Medical and Health Sciences, Kesmonds International University, Mile 3 Nkwen, Bamenda, Cameroon; 4 Department of Basic Sciences, Faculty of Dentistry, Damascus University, Damascus, Syria; 5 Key Laboratory of Organic Industries, Department of Chemistry, Faculty of Sciences, Damascus University, Damascus, Syria; 6 Faculty of Medicine and Pharmaceutical Sciences, University of Douala, Douala, Cameroon; 7 Department of Pure and Industrial Chemistry, Abia State University, Uturu, Nigeria; 8 Department of Public Health, Faculty of Medicine and Pharmaceutical Sciences, University of Dschang, Dschang, West Region Cameroon; 9 Faculty of Health Sciences, University of Bamenda, Bamenda, North West Region Cameroon; 10 Department of Obstetrics/Gynaecology and Maternal Health, Faculty of Medicine and Pharmaceutical Sciences, University of Dschang, Dschang, West Region Cameroon

**Keywords:** Biochemistry, Endocrinology, Medical research, Risk factors

## Abstract

Multiple electrolyte disorders, including sodium, potassium and calcium disorders, have been associated with hypertension in pregnancy. Most of these studies failed to evaluate the combined effect of low and high sodium, potassium, calcium and chloride ion concentrations on hypertension in pregnancy. This study evaluates the combined effect of these ion categories (low, normal, high) on hypertension in pregnancy. Biochemical ion assays and blood pressure measurements were carried out on 1074 apparently healthy pregnant women in late third trimester. Serum potassium, sodium, chloride, and ionised calcium were measured by ion-selective electrode potentiometry, while total plasma calcium was measured by absorption spectrophotometry. Hypertension in pregnancy was defined as systolic blood pressure ≥ 140 mmHg and/or diastolic blood pressure ≥ 90 mmHg. The prevalence of hyponatraemia, hypokalaemia, hypochloraemia, ionised hypocalcaemia and total hypocalcaemia in late pregnancy was 1.30 [0.78–2.18]%, 3.55 [2.60–4.84]%, 1.96 [1.28–2.97]%, 1.49 [0.92–2.21]% and 43.58 [40.64–46.56]%, respectively. Hypernatraemia, hyperkalaemia, hyperchloraemia, ionised hypercalcaemia and total hypercalcaemia were found in 1.49 [0.92–2.41]%, 2.34 [1.59–3.43]%, 4.38 [3.31–5.77]%, 39.94 [37.06–42.90]%, 2.79 [1.96–3.96]% of the participants, respectively. The prevalence of hypertension in pregnancy was 7.17 [5.77–8.87]%. When ion categories were considered in multiple logistic regression, only ionised and total calcium had significant associations with hypertension in pregnancy. Women with ionised hypercalcaemia had lower odds of hypertension in pregnancy (AOR = 0.50 [0.29–0.87], p-value = 0.015), and women with total hypocalcaemia had higher odds of hypertension in pregnancy (AOR = 1.99 [1.21–3.29], p-value = 0.007), compared to women with ionised and total normocalcaemia, respectively. Increasing kalaemia was associated significantly with higher odds of hypertension in pregnancy; however, kalaemia below and above the normal concentrations had no significant association with hypertension. Nonetheless, participants with kalaemia ≤ 3.98 mmol/L, had lower odds of hypertension in pregnancy compared with those with higher kalaemia (OR = 0.40 [0.24–0.66], p-value = 0.0003). Calcium disorders remain the most frequent electrolyte disorders in pregnancy. When normal cut-offs are considered for calcium and other ions, only ionised and total calcium influence the occurrence of hypertension in pregnancy. Kalaemia seems to affect hypertension in pregnancy but primarily within its normal concentrations. Serum electrolyte follow-up is indispensable for a proper pregnancy follow-up.

## Introduction

Serum electrolytes play an indispensable role in controlling and maintaining human physiology. Ion concentrations in blood and the internal milieu must be kept within a specific range for the body systems to continue running efficiently. Deviations from normal concentrations are usually associated with physiological malfunctions, leading to the occurrence of some symptoms^1^.

Hyponatraemia can occur in pregnancy and is associated with some pregnancy outcomes, which usually depend on the speed of drop of sodium concentrations^2,3^. While its most common cause in the first trimester remains excessive vomiting (hyperemesis gravidarum)^4^, in the second half of pregnancy, it is usually due to pre-eclampsia. Low natraemia is associated with twin pregnancy, significant oedema in the woman and early onset of pre-eclampsia^5^. It has also been highly correlated to the severity of pre-eclampsia^2^. Given that transfers from foetus to mother can occur across the placenta to correct maternal hyponatraemia, neonatal hyponatraemia usually occurs with tachypnoea, seizures, jaundice, and neonatal intensive care admission^6,7^. Literature on hypernatraemia in pregnancy is practically non-existent. Natraemia has been described to drop by 3-5 mmol/l with an associated reduction of osmolality of 10 mOsm/kg in normal pregnancy^1,2^.

In a nationwide study on the prevalence and risk factors of hypokalaemia in the United States, only 0.69% of pregnant women had hypokalaemia. Younger age, low-income level, and being of the black race were significantly associated with higher odds of hypokalaemia^8,9^. In South Africa, the prevalence of hypokalaemia has been reported to be as high as 5.3%^8^. Hypokalaemia in pregnancy is associated with hyperemesis gravidarum, gestational hypertension and postpartum haemorrhage^9^. On the other hand, hyperkalaemia has not been described in normal cases of pregnancy. Most of the cases in pregnancy have been reported in association with magnesium sulphate therapy^10,11^ and kidney disease^12^. Besides causes linked to drug intake, nutrition during pregnancy plays a major role in exposing women to either low or high kalaemia^13^.

Chloride ions contribute to the regulation of body fluids, keeping electrolyte balance, preserving electrical neutrality, and acid–base equilibrium. Studies focused on deviations from normal blood chlorine concentrations, and their consequences are scarce, especially in pregnancy^14^.

The prevalence of total hypocalcaemia in pregnancy has been reported to be high. Studies in the third-trimester of pregnancy have recorded the prevalence of total hypocalcaemia to be between 58 and 70%^15–18^. However, the prevalence of ionised hypocalcaemia in pregnancy during the third trimester has been found to be as low as 2.98%^18^. Low calcium intake and hypocalcaemia in pregnancy have been associated with multiple adverse maternofoetal outcomes ranging from hypertensive diseases in pregnancy (pre-eclampsia), poor Apgar scores, small for gestational age, low bone mass, and short birth length^19,20^. However, hypertensive diseases in pregnancy have been described to be more associated with ionised calcium concentrations than total calcium concentrations^20,21^.

Hypercalcaemia in pregnancy is an underdiagnosed complication capable of causing adverse maternal and foetal outcomes. Its underdiagnosis is due to its asymptomatic nature or due to the fact that, its symptoms like fatigue or nausea usually mimic symptoms of early pregnancy and emesis gravidarum^22^. Its major cause in more than 90% of cases is primary hyperparathyroidism, newly diagnosed in pregnancy^22,23^. Chronic hypercalcemia in pregnancy can lead to pre-eclampsia, nephrolithiasis, and pancreatitis in the mother^24^. It is associated with foetal growth restriction, severe neonatal hypocalcaemia, tetany, and death due to fetal hypoparathyroidism^22,24–27^.

Hypertensive disorders in pregnancy, particularly pre-eclampsia/eclampsia, remain among the leading causes of maternal mortality worldwide. They complicate about 8–10% of pregnancies, are associated with severe adverse maternofoetal outcomes, and contribute to a significant proportion of perinatal deaths^28–30^. Abnormalities in calcaemia, kalaemia, and natraemia have been associated with hypertensive disorders in pregnancy, principally pre-eclampsia^31–36^. However, most of these studies, instead of using normal ion cut-offs to directly evaluate the effect on dichotomised blood pressure values (high or normal blood pressure), compare mean ion concentrations between groups (hypertensives and normotensives). This makes it difficult to assess the combined effect of multiple ion abnormalities on blood pressure in pregnancy. Moreover, some of these studies have ended with only correlation analysis between blood pressure values and ion concentrations, without a clear definition of the effects of abnormal ion concentrations. Multiple studies have been conducted to find the effect of total calcaemic states on blood pressure in pregnancy. A few have focused on the contribution of ionised calcaemia^20,21^, while studies which have tried to evaluate the combined effect of calcium, sodium, potassium and chloride ions in maintaining blood pressure are very scarce. This study was therefore designed to evaluate the effect of the deviation of different ion concentrations from the normal range on hypertension in pregnancy.

## Methodology

### Study design

This was a cross-sectional hospital-based study targeting term pregnant women (At least 37 weeks of pregnancy) in the Nkongsamba Health District. Data were obtained from biochemical blood assays, and analysis was done using the statistical software Epi-Info, version 7.2.5.0.

### Study setting

This study was conducted in the Nkongsamba Health District, Littoral Region of Cameroon. It is one of the largest Health Districts of the Moungo Division and covers most of the Nkongsamba 1, 2 and 3 sub-divisions. The four top health facilities of the Health District, which receive and manage 70–90% of pregnant women in the district, were selected for the study. These included the Nkongsamba Regional Hospital (the main referral hospital of the Health District), the Catholic Medicalised Health Centre, the Fultang Polyclinic, and the Good Samaritan Medicalised Health Centre. All the data were collected from term pregnant women in the maternities of the four selected health facilities.

### Study population and eligibility criteria

Our study population included all pregnant women in the Nkongsamba Health District. However, only apparently healthy pregnant women (at least 37 complete weeks of pregnancy) received in the four selected health facilities for routine antenatal care were included in the study.

### Sampling and sample size

Based on the prevalence of total hypocalcaemia (the most common serum ion abnormality recorded so far, and capable of having more significant adverse maternal and foetal outcomes), we estimated the sample size for the study. Using Cochran’s formula, an expected proportion of women with hypocalcaemia in the third trimester of 59%, an acceptable two-sided alpha error of 5%, and the absolute precision on either side of the proportion of 0.03, we estimated a minimum required sample size to 1033 participants. Consecutive recruitment of participants was carried out at the level of the four health facilities.

### Procedure of implementation

Administrative authorisations were obtained from the District Medical Officer of the Nkongsamba Health District and the Directors of the different health facilities. The ethical clearance for the study was obtained from the Cameroon Bioethics Initiative/Ethics Review and Consultancy Committee. Midwives working in these selected maternities were trained on the informed consent process, data collection procedure and blood sample collection. The collection of blood samples and measurement of the participants' blood pressure were done during the same visit. Eligible and consenting pregnant women were allowed to rest in a sitting and relaxed position for at least 10 min before any data were collected.

### Collection of blood samples

The participant was allowed to relax and breath calmly for about 5 min. Participants were asked not to exercise the forearm in anyway before sample collection and fist formation was not allowed during sample collection. As per the World Health Organisation guidelines of best practice for phlebotomy, blood samples were carefully collected using evacuated needles, with tourniquets placed for less than a minute^37^. About 10 ml of blood was collected into a dry vacutainer tube, settled for 30 min, and the serum was used to measure chlorine, potassium, sodium and ionised calcium concentrations in blood^38,39^. Also, 10 ml of blood was drawn into lithium-heparinised tubes and used to measure total calcaemia^40^. For collection into heparinised tubes, each tube was filled to its maximum capacity, mixed slowly and surely with the anticoagulant. All tubes were kept sealed and handled anaerobically during the biochemical assays.

### Measurement of blood pressure

Each participant's blood pressure was measured immediately after collecting the blood sample. After 10 min of rest in a sitting position, the brachial blood pressure was taken using an aneroid sphygmomanometer and an adapted cuff. The adapted cuff was fastened on the bare skin of the arm, around mid-point of the sternum, with the participant’s arm resting on the examination table at heart level. For each arm, two blood pressure measurements were taken at a 2 min interval, and the average for the diastolic and systolic values calculated. The recorded blood pressure was taken from the arm with the highest mean blood pressure values^41^. Hypertension in pregnancy (the major outcome in this study) was defined using the World Health Organisation cut-offs for systolic and diastolic hypertension (systolic blood pressure ≥ 140 mmHg and/or diastolic blood pressure ≥ 90 mmHg)^29^.

### Biochemical assays

For each participant, ionised calcium, sodium, potassium, and chloride ion concentration were measured in serum using ion-selective electrode potentiometry. These were measured using the K-Lite8 Series Advanced Blood Gas Electrolyte Analyser (MSLEA15-H model), and its reagent kits and solutions (calibration solutions A and B, and solutions for electrode maintenance), manufactured in Guangzhou Medsinglong Medical Equipment Co., Ltd, china. The apparatus is equipped with an electrode for ionised calcium, sodium, potassium, chlorine, pH and a reference electrode. All measurements were carried out following standard operating procedures for the apparatus. Ionised calcium concentrations were then corrected for pH changes as per this equation: Corrected iCa^2+^ (pH 7.4) = Measured iCa^2+^ [1 − 0.53 × (7.40 − measured pH)], which is valid between 7.2 and 7.6^42^.

Total calcaemia was measured in plasma by absorption spectrophotometry. Blood collected in lithium heparinised tubes was centrifuged, and the plasma extracted for the measurement of total calcium. In this process, the semi-automatic KENZA MAX BioChemis Try Analyser with BIOLABO standard reagent packages was used. Total calcium concentrations were measured using the o-cresolphthalein complexone method method, and all measurements were done per BIOLABO standard operating procedures^43^.

Low and high ion concentrations were defined based on the standard cut-offs for normal concentrations in the third trimester of pregnancy^44^. See Table 1 below.

**Table 1:** Normal range for some serum electrolytes in the third trimester of pregnancy.

Ion	The normal range in the third trimester of pregnancy
Sodium (mEq/L or mmol/L)	130–148
Potassium (mEq/L or mmol/L)	3.3–5.1
Chlorine (mEq/L or mmol/L)	97–109
Ionised calcium (mg/dL)	4.4–5.3
Total calcium (mg/dL)	8.2–9.7

This table is drafted from reference number^44^.

### Data analysis

All research data were entered into a predesigned data entry sheet on Epi-Info, version 7.2.5.0 and analysed. Based on normal cut-offs for each ion measured, participants were categorised into low, normal and high ion concentrations. Hypertension in pregnancy (the major outcome in this study) was defined as systolic blood pressure ≥ 140 mmHg and/or diastolic blood pressure ≥ 90 mmHg. Proportions and their 95% confidence intervals were then calculated for each ion category as a percentage. Means and standard deviations were also estimated for each ion concentration. The strength of the association between hypertension in pregnancy (the outcome of interest) and each of the ion categories was measured using the odds ratio and its 95% confidence interval in simple logistic regression. The combined effect of the categories of all the ions on the outcome of interest was evaluated in a multiple logistic regression model. The statistical significance threshold for each analysis was set at 5%.

### Ethical considerations

All methods were carried out in accordance with relevant guidelines and regulations. Informed consent was obtained from all subjects and/or their legal guardians. Only consenting women were included in this study. Participants signed consent forms before any data collection was carried out. For minors, apart from signing assent forms, their legal representatives signed consent forms for them. Participant’s confidentiality was respected by questionnaire coding and preservation of research questionnaires within the research team. In addition, questionnaires were burned after data had been entered into the database. Ethical clearance for the study was obtained from the Cameroon Bioethics Initiative/Ethics Review and Consultancy Committee.

## Results

### Characteristics of study participants

Data for analysis was collected from 1074 women in late third trimester. The age range of the participants was 15–47 years, with a mean age of 28.12 ± 6.08 years. Table 2 summarises a few characteristics of the participants. Participants were mostly aged between 21 and 30 years (56.52%), while 47.06% of these women were between their second and fourth pregnancies.

**Table 2:** Characteristics of the participants.

Characteristic	Modalities	Frequency	Proportions (%)
Age groups (n = 1074)	15–20 years	97	09.03
	21–30 years	607	56.52
	31–50 years	370	34.45
Marital status (n = 1068)	Single	371	34.74
	Married	269	25.19
	Cohabiting	462	39.89
	Widow	02	0.19
Level of education (n = 1074)	Primary	72	06.60
	Secondary	741	68.99
	Higher	261	24.30
Number of pregnancies (n = 1071)	First pregnancy	256	23.90
	2–4	504	47.06
	5–8	259	24.18
	More than 8	51	04.76
Number of deliveries (n = 1067)	None	249	23.34
	1–3	596	55.86
	4–6	205	19.21
	More than 6	17	01.59

### Range and mean concentrations of the different ions

The range and mean concentrations of the ions are shown in Table 3 below. The mean concentrations of all the ions were within the normal range reported in literature. However, there were deviations on either side of each range in comparison to literature.

**Table 3:** Range and mean concentrations of the different ions measured.

Ion	Range (mmol/L) [normal range]	Mean concentration ± standard deviation (mmol/L)
Sodium (n = 1074)	117.00–163.00 [130–148]	140.66 ± 4.30
Potassium (n = 1069)	02.42–6.00 [3.3–5.1]	03.98 ± 0.47
Chloride (n = 1074)	82.00–119.00 [97–109]	102.79 ± 3.57
pH-corrected ionised calcium (n = 1074)	0.71–01.65 [1.1–1.33]	01.31 ± 0.08
Total calcium	01.25–02.47 [2.05–2.43]	02.05 ± 0.22

### Prevalence of hypertension in pregnancy, low and high ion concentrations in blood

Among the 1074 pregnant women included in the study, 77 (7.17 [5.77–8.87]%) presented with hypertension in pregnancy. The prevalence of low and high ion concentrations is presented in Table 4. For sodium, potassium and chloride ions, the prevalence of serum abnormalities was low when considered from reported cut-off values. The prevalence of hyponatraemia and hypernatraemia was 1.30 [0.78–2.18]% and 1.49 [0.92–2.41]%, respectively. That of hypokalaemia (3.55 [2.60–4.84]%) was higher than that of hyperkalaemia (2.34 [1.59–3.43]%). The study recorded more cases of hyperchloraemia (4.38 [3.31–5.77]) than hypochloraemia (1.96 [1.28–2.97]).

**Table 4:** Prevalence of low and high ion concentrations among the participants.

Ions	Prevalence of low, normal and high ion concentrations (%)			
	Modalities	Hypertension (N = 77)	No hypertension (N = 997)	Total prevalence (95% confidence interval)
Sodium (N = 1074)	Low (n = 14)	01 (01.30)	13 (01.30)	01.30 (0.78–2.18)
	Normal (n = 1044)	75 (97.40)	969 (97.19)	97.21 (96.04–98.04)
	High (n = 16)	01 (1.30)	15 (01.50)	01.49 (0.92–2.41)
Potassium (N = 1069)	Low (n = 38)	03 (03.95)	35 (03.52)	03.55 (2.60–4.84)
	Normal (n = 1006)	70 (92.1)	936 (94.26)	94.11 (92.53–95.37)
	High (n = 25)	03 (03.95)	22 (02.22)	02.34 (1.59–3.43)
Chloride (N = 1074)	Low (n = 21)	02 (02.60)	19 (01.91)	01.96 (1.28–2.97)
	Normal (n = 1006)	70 (90.91)	936 (93.88)	93.67 (92.05–94.98)
	High (n = 47)	05 (06.49)	42 (04.21)	04.38 (3.31–5.77)
pH-corrected ionised calcium (N = 1074)	Low (n = 16)	02 (02.60)	14 (01.40)	01.49 (0.92–2.41)
	Normal (n = 629)	57 (74.03)	572 (57.37)	58.57 (55.59–61.48)
	High (n = 429)	18 (23.38)	411 (41.22)	39.94 (37.06–42.90)
Total calcium (N = 1074)	Low (n = 468)	46 (59.74)	422 (42.33)	43.58 (40.64–46.56)
	Normal (n = 576)	30 (38.96)	546 (54.76)	53.63 (50.64–56.60)
	High (n = 30)	01 (01.30)	29 (02.91)	02.79 (1.96–3.96)

As concerns ionised and total calcaemia, significant abnormalities were noted. Using the standard reported cut-off values, only 58.57% of these women had normal ionised calcaemia. While only 1.49 [0.92–2.41]% had low ionised calcaemia, and up to 39.94 [37.06–42.90]% had values classified as high. The prevalence of ionised hypocalcaemia among hypertensive pregnant women was higher than its prevalence among normotensive pregnant women. However, the prevalence of ionised hypercalcaemia among normotensive pregnant women was higher than its prevalence among hypertensive pregnant women. Total hypocalcaemia (43.58 [40.64–46.56]%) was very frequent compared to total hypercalcaemia (2.79 [1.96–3.96]%) in the third trimester. The prevalence of total hypocalcaemia among hypertensive pregnant women was higher than its prevalence among normotensive pregnant women. Moreover, total calacemia and ionised calcaemia correlated positively (*r* = 23.59, r^2^ = 0.04, p-value = 0.0000).

### Association of ion concentrations with hypertension in pregnancy

The association between different categories of ion concentrations and hypertension in pregnancy is presented in Table 5. Among all the ions tested, only ionised and total calcaemia had a statistically significant association with hypertension in pregnancy.

**Table 5:** Simple logistic regression between hypertension in pregnancy and different ion categories.

Ion	Ion concentration	Hypertension (N = 77)	No hypertension (N = 997)	Odds ratio	95% confidence interval	p-value
Sodium	Low (n = 14)	01	13	0.99	0.13–7.70	0.995
	Normal* (n = 1044)	75	969			
	High (n = 16)	01	15	0.86	0.11–6.61	0.886
Potassium	Low (n = 38)	03	35	1.15	0.34–3.82	0.824
	Normal* (n = 1006)	70	936			
	High (n = 25)	03	22	1.82	0.53–6.24	0.339
Chloride	Low (n = 21)	02	19	1.41	0.32–6.17	0.650
	Normal* (n = 1006)	70	936			
	High (n = 47)	05	42	1.59	0.61–4.15	0.342
pH-corrected ionised Calcium	Low (n = 16)	02	14	1.43	0.32–6.46	0.639
	Normal* (n = 629)	57	572			
	High (n = 429)	18	411	0.44	0.25–0.76	0.003**
Total calcium	Low (n = 468)	46	422	1.98	1.21–3.20	0.005**
	Normal* (n = 576)	30	546			
	High (n = 30)	01	29	0.63	0.08–4.76	0.653

Prevalence of hypertension in pregnancy: 77/1074 = 07.17%

*Reference.

**p-value < 0.05: Statistically significant.

Women with ionised hypercalcaemia had 0.44-fold lower odds for hypertension in pregnancy compared to their counterparts with ionised normocalcaemia (OR = 0.44 [0.25–0.76], p-value = 0.003). Also, pregnant women with total hypocalcaemia had 1.98-fold higher odds for hypertension in pregnancy compared with their counterparts with total normocalcaemia (OR = 1.98 [1.21–3.20], p-value = 0.005).

Table 6 shows the direction of associations of crude ion concentrations with hypertension in pregnancy in a multiple logistic regression model. All the ions had a negative association with hypertension in pregnancy except for potassium. However, significant associations were recorded only for potassium and ionised calcium. The odds of hypertension in pregnancy significantly increased with increasing kalaemia and decreasing calcaemia.

**Table 6:** Multiple logistic regression between crude ion concentrations and hypertension in pregnancy.

Ion	Adjusted odds ratio	95% confidence interval	p-value
Sodium	0.99	0.94–1.04	0.583
Potassium	2.47	1.59–3.84	0.0001
Chloride	0.96	0.90–1.02	0.242
Ionised calcium	0.01	0.0005–0.1314	0.0007
Total calcium	0.98	0.95–1.00	0.084

Table 7 shows the association between hypertension in pregnancy and different ion categories following multiple logistic regression. The relationship between ionised calcaemia, total calcaemia and hypertension in pregnancy persisted. Women with ionised hypercalcaemia had a 0.50-fold lower odd for hypertension in pregnancy compared with their counterparts with normal calcaemia (AOR = 0.50 [0.29–0.87], p-value = 0.015). Also, women who had total hypocalcaemia had a 1.99-folds higher odd of hypertension in pregnancy compared with their counterparts with normal total calcaemia (AOR = 1.99 [1.21–3.29], p-value = 0.007).

**Table 7:** Multiple logistic regression between hypertension in pregnancy and different ion categories.

Ion	Ion category	Hypertension (N = 77)	No hypertension (N = 997)	Odds ratio	95% confidence interval	p-value
Sodium	Low (n = 14)	01	13	0.93	0.09–9.61	0.954
	Normal* (n = 1044)	75	969			
	High (n = 16)	01	15	0.57	0.06–5.16	0.614
Potassium	Low (n = 38)	03	35	0.97	0.28–3.39	0.958
	Normal* (1006)	70	936			
	High (n = 25)	03	22	2.31	0.63–8.48	0.205
Chloride	Low (n = 21)	02	19	0.85	0.16–4.64	0.852
	Normal* (n = 1006)	70	936			
	High (n = 47)	05	42	1.68	0.60–4.70	0.323
pH-corrected ionised calcium	Low (n = 16)	02	14	1.28	0.25–6.63	0.772
	Normal* (n = 629)	57	572			
	High (n = 429)	18	411	0.50	0.29–0.87	0.015**
Total calcium	Low (n = 468)	46	422	1.99	1.21–3.29	0.007**
	Normal* (n = 576)	30	546			
	High (n = 30)	01	29	0.59	0.07–4.61	0.613

Prevalence of hypertension in pregnancy: 77/1074 = 07.17%

All ions and their categories were included in the multiple logistic regression model.

*Reference.

**p-value < 0.05: Statistically significant.

When uncategorised kalaemia is used with hypertension in pregnancy, an association is noted. However, when categorised based on normal cut-offs for kalaemia in the third trimester, no significant associations are observed. This suggests that the effect of kalaemia on blood pressure in pregnancy might occur within the normal range of kalaemia. When the kalaemia was bisected around the mean concentration in this study (03.98 mmol/L), participants with kalaemia ≤ 3.98 mmol/L, were found to have a 0.40-fold lower odd of hypertension in pregnancy compared with those with higher kalaemia (OR = 0.40 [0.24–0.66], p-value = 0.0003).

## Discussion

Serum electrolyte concentrations have been studied in multiple pathological and normal conditions in humans. According to the results of this study, serum electrolyte concentrations in pregnancy are liable to experience changes which sometimes go below or beyond the normal concentrations. The most common variations were observed with calcium compared with other ions. Variations of blood ion concentrations in pregnancy do not only affect maternal physiology, but go a long way to impact foetal outcomes.

In our study population, the prevalence of hyponatraemia (01.30 [0.78–2.18]) and hypernatraemia (01.49 [0.92–2.41]) in late third trimester was found to be low. This implies that only about one to two in a hundred women in late third-trimester experience hyponatraemia or hypernatraemia. The prevalence of hyponatraemia in pregnancy has not been clearly stated in previous studies, and most of these studies have focused on the association of hyponatraemia with pre-eclampsia. Moreover, it is most often reported in the setting of hyperemesis gravidarum, which occurs mainly during the first trimester^3,4^. In a study on the outcomes associated with hyponatraemia in pregnancy, hyponatraemia was reported in 0.27% of all deliveries, with up to 9% of cases of pre-eclampsia associated with hyponatraemia^2^. To the best of our knowledge, hypernatraemia in pregnancy has not been reported in literature. The current study found that a few pregnant women can experience hypernatraemia. This could be due to insufficient fluid intake in pregnancy, excessive loss of water or subclinical severe conditions like kidney malfunction and diabetes^45^. However, our study failed to further investigate this group of women.

Hypokalaemia in pregnancy has also received some research interest. The prevalence of hypokalaemia in pregnancy reported in our study was 3.55%, which seems to be about 3–4 times higher than the reported prevalence in developed nations. Studies in the United States have reported the prevalence of hypokalaemia in pregnancy to be around 1%^9,46^. However, in a nationwide study carried out in the United States, younger age and black race were more exposed to hypokalaemia compared with their counterparts^9^. In South Africa, the prevalence of hypokalaemia in pregnancy was reported to be 5.3%^8^. Our results are in line with suggestions that the prevalence of hypokalaemia in pregnancy among blacks (Africans) might be significantly higher. Even though not evaluated in this study, hypokalaemia can be due to excessive potassium loss from the gastrointestinal tract (vomiting or diarrhoea),chronic kidney injury, excessive sweating, and diabetes^47^. On the other hand, hyperkalaemia in pregnancy was reported in 2.34% of the study population. This prevalence seems to be high compared to expectations in normal pregnancy in the third trimester. Most cases of hyperkalaemia in pregnancy have been associated with kidney injuries^12^ and complications of magnesium sulphate therapy in the context of pre-eclampsia^10,11^.

In this study, 1.96% of these women had hypochloraemia in pregnancy, and up to 4.38% had hyperchloraemia in pregnancy. No existing studies were found in literature for comparison. However, we feel that this suggests that in the same light as natraemia and kalaemia in pregnancy, chloraemia is liable to vary in situations of pregnancy.

Using published cut-offs for normal ionised and total calcaemia^44^, the highest proportion of women with ion concentration abnormalities was found to concern calcium. Only a little more than 50% of the women had normal ionised and total calcaemia at the time of the study. The relatively high prevalence of total hypocalcaemia in this study has been reported in similar studies^15,16,48^. The slightly lower prevalence of total hypocalcaemia could be explained by the lower cut-off used to defined hypocalcaemia in this study. Total hypercalcaemia was found in 2.79% of these women. According to literature, hypercalcaemia in pregnancy is underdiagnosed, and no studies have evaluated its prevalence^22^. The diagnosis of total hypercalcaemia in pregnancy should warrant a hormonal workup for possible hyperparathyroidism in pregnancy^22,23^.

Ionised hypocalcaemia is rare in pregnancy. However, based on normal cut-offs, up to 39.94% of the study population was classified as having ionised hypercalcaemia. The mean ionised calcium concentration in this study (01.31 ± 0.08 mmol/L) was found to be very close to the upper limit of the normal range in the third trimester of pregnancy (1.33 mmol/L). In this study, data were collected among apparently healthy pregnant women, and the measured ionised calcium concentrations were corrected for pH changes before analysis. In literature, no studies have been conducted to evaluate the prevalence of ionised hypercalcaemia in pregnancy and its risk factors. If the published upper limit of normal ionised calcaemia is correct, the prevalence of ionised hypercalcaemia in late pregnancy is likely high. Despite the positive correlation between total and ionised calcaemia, the high prevalence of total hypocalcaemia is not associated with a high rate of ionised hypocalcaemia. Instead, the rate of ionised hypercalcaemia seemed to be relatively high. Nonetheless, it is possible that pregnant women in late pregnancy turn to develop and tolerate relatively higher concentrations of ionised calcaemia, despite the high rates of total hypocalcaemia they experience. This phenomenon requires a more sophisticated study that follows variations of ionised calcaemia from the first trimester to the third trimester. This is likely going to lead to a review of the normal ionised calcium concentrations for the different trimesters.

Hypertension in pregnancy remains a serious problem associated with adverse maternal and foetal outcomes. From the results of this study, when all crude ions were included in a multiple logistic model, ionised calcaemia and kalaemia showed statistically significant associations with hypertension in pregnancy. Higher ionised calcaemia was significantly associated with lower odds of hypertension in pregnancy. This direction of association has been reported in other studies^21,31^. However, increasing kalaemia was associated with increased odds of hypertension in pregnancy. This is contrary to findings stating that women with hypertension in pregnancy (or with pre-eclampsia) had a significantly lower mean kalaemia than those who were normotensive^32,36^. However, studies have also found no statistically significant differences in mean kalaemia between normotensive and hypertensive pregnant women^33,35^. Notwithstanding, categorising kalaemia into high, normal and low categories showed that these categories did not have any statistically significant association with hypertension in pregnancy. This implies that kalaemia might have an effect on the likelihood of hypertension in pregnancy, but this occurs mainly within the normal range of kalaemia in pregnancy. Bisecting kalaemia in these women around the mean showed that women with higher kalaemia (above the mean) had significantly higher odds of hypertension in pregnancy compared to women with lower kalaemia.

Following multiple logistic regression in a test for the association of different ion categories with hypertension in pregnancy, ionised calcaemia and total calcaemia were significantly associated with hypertension in pregnancy. Multiple studies have found the mean ionised calcium concentrations to be significantly lower among hypertensive pregnant women compared to normotensive pregnant women^21,31,35^. Our study also found the prevalence of ionised hypercalcaemia to be higher among women with normal blood pressure compared to hypertensive pregnant women. Also, mean total calcium levels have also been found to be higher among normotensive pregnant women compared with hypertensive pregnant women^21^. Our findings suggest that even higher concentrations of ionised calcaemia in pregnancy are associated with better blood pressure values. It is likely that ionised calcaemia values above normal concentrations might be beneficial in reducing blood pressure in pregnancy. However, much work needs to be carried out in following up the variation of ionised calcaemic states through the different trimesters in pregnancy.

In conclusion, apart from ionised calcium and total calcium concentrations, falling out of the normal range of different ions (sodium, potassium, chloride) does not significantly impact the likelihood of hypertension in pregnancy.

The interpretation of these results should consider that these are analyses from an instantaneous measurement of serum electrolytes. Apart from missing or picking up some changes in electrolyte concentrations, which might only be acute, we did not consider the influence of other ions like magnesium ions and bicarbonates. Our study failed to justify serum electrolyte deviations which require evaluation of hormonal changes and variation of some biochemical indicators like serum creatinine, follistatin, renal function, fasting glucose, fasting lipid panel, liver enzymes, and uric acid. Moreover, our study only established associations and not causal relationships. Hypertension in pregnancy was also considered a block in this study. It should, therefore, not be directly interpreted as pre-eclampsia or gestational hypertension.

## Conclusion

The prevalence of high and low natraemia, kalaemia and chloraemia are very low in late pregnancy. Ionised hypocalcaemia is also a rare finding in pregnancy. Despite the positive correlation between ionised calcaemia and total calcaemia, relatively high rates of total hypocalcaemia and ionised hypercalcaemia occur in pregnancy. Changes in kalaemia within its normal range in pregnancy are likely to influence the likelihood of hypertension in pregnancy. Apart from ionised and total calcaemia, falling out of the normal range of different ions (sodium, potassium, chloride) has no significantly influence on the likelihood of hypertension in pregnancy. Practitioners should be encouraged to carry out routine serum electrolyte profile follow-up in pregnancy to detect and manage any severe deviations which can affect foetal outcomes. More research is required to explore and define the normal ionised calcaemia range in the third trimester of pregnancy.

## Data Availability

All datasets analysed in this manuscript are fully available from corresponding author on reasonable request.
